# Assessment of sleep patterns in dementia and general population cohorts using passive in-home monitoring technologies

**DOI:** 10.1038/s43856-024-00646-0

**Published:** 2024-10-31

**Authors:** Louise Rigny, Nan Fletcher-Lloyd, Alex Capstick, Ramin Nilforooshan, Payam Barnaghi

**Affiliations:** 1 Department of Brain Sciences, Imperial College London, London, UK; 2 Great Ormond Street Hospital, London, UK; 3 UK Dementia Research Institute, Care Research and Technology Centre, London, UK; 4 Surrey and Borders Partnership NHS Foundation Trust, Leatherhead, UK; 5 University of Surrey, Guildford, UK

**Keywords:** Dementia, Disease prevention, Medical research

## Abstract

**Background:**

Nocturnal disturbances are a common symptom experienced by People Living with Dementia (PLWD), and these often present prior to diagnosis. Whilst sleep anomalies have been frequently reported, most studies have been conducted in lab environments, which are expensive, invasive and not natural sleeping environments. In this study, we investigate the use of in-home nocturnal monitoring technologies, which enable passive data collection, at low cost, in real-world environments, and without requiring a change in routine.

**Methods:**

Clustering analysis of passively collected sleep data in the natural sleep environment can help identify distinct sub-groups based on sleep patterns. The analysis uses sleep activity data from; (1) the Minder study, collecting in-home data from PLWD and (2) a general population dataset (combined *n* = 100, >9500 person-nights).

**Results:**

Unsupervised clustering and profiling analysis identifies three distinct clusters. One cluster is predominantly PLWD relative to the two other groups (72% ± 3.22, *p* = 6.4 × 10^−7^, *p* = 1.2 × 10^−2^) and has the highest mean age (77.96 ± 0.93, *p* = 6.8 × 10^−4^ and *p* = 6.4 × 10^−7^). This cluster is defined by increases in light and wake after sleep onset (*p* = 1.5 × 10^−22^, *p* = 1.4 × 10^−7^ and *p* = 1.7 × 10^−22^, *p* = 1.4 × 10^−23^) and decreases in rapid eye movement (*p* = 5.5 × 10^−12^, *p* = 5.9 × 10^−7^) and non-rapid eye movement sleep duration (*p* = 1.7 × 10^−4^, *p* = 3.8 × 10^−11^), in comparison to the general population.

**Conclusions:**

In line with current clinical knowledge, these results suggest detectable dementia sleep phenotypes, highlighting the potential for using passive digital technologies in PLWD, and for  detecting architectural sleep changes more generally. This study indicates the feasibility of leveraging passive in-home technologies for disease monitoring.

## Abstract (web summary)

Rigny et al. evaluate passive in-home nocturnal monitoring in real-world settings to identify distinct sleep pattern sub-groups between people living with dementia and the general population. The feasibility of using these technologies for screening dementia-related sleep signatures and monitoring broader sleep changes is demonstrated.

## Plain language summary

People living with dementia commonly sleep poorly at night, and this often occurs before they are diagnosed with dementia. We investigated whether a sleep sensor placed under a person’s mattress could monitor sleep activity in people with dementia without disrupting their normal daily routines and behaviour. We compared sleep data collected from both people with dementia and the general population to identify whether differences could be detected. We found identifiable dementia-related sleep patterns, suggesting sleep sensors could be used both to monitor disease and more generally in research. In the future, using these types of sensors could enable better care for people living with dementia by monitoring their sleep.

## Introduction

Approximately 1 million people are thought to be living with dementia in the UK, and over 55 million worldwide, representing the leading cause of disability in older adults^1,2^. Dementia is characterised by a progressive decline in cognitive and functional abilities, affecting numerous faculties, such as memory, behaviour, and executive functioning^3^. Presently, treatment is limited. Current treatments focus on symptom management and addressing already established health conditions that affect people living with dementia (PLWD), and their effectiveness relies on early intervention. As such, current clinical guidelines focus on timely detection^4^. However, identifying dementia symptoms early can be difficult, due to dementia’s insidious onset. Additionally, PLWD may accommodate or compensate for their symptoms earlier on.

Nocturnal disruptions are one of the most commonly experienced symptoms of dementia. A growing body of evidence indicates that sleep disorders may act as a predictor of dementia incidence, such as mild cognitive impairment (MCI)^5,6^, where alterations in sleep quality may generate and/or accelerate the rate of cognitive decline, both in the presence and absence of pathology^7,8^. Indeed, those with poorer sleep have been shown to have poorer outcomes, including having an increased risk of developing cerebrovascular pathology and a worse dementia prognosis^9,10^.

Whilst evidence indicates sleep disturbances contribute to dementia progression, the exact reasons for changes in sleep architecture is unclear. It is plausible that changes originate from Alzheimer’s disease (AD) pathology-induced neuronal and synaptic damage^11^. In AD, degeneration occurs in regions where sleep centres reside, such as the basal forebrain, hypothalamus, thalamus, midbrain, pons, and the brainstem^12^. A previous study also reported a loss of galanin-positive neurons in the ventrolateral preoptic region in individuals with AD, where the decrease positively correlated with an increased sleep fragmentation^13^. Conversely, reductions in Non-Rapid Eye Movement (NREM) sleep were associated with tau pathology prior to cognitive decline in early AD patients^14^. Additionally, over 80% of those with Rapid Eye Movement (REM) sleep behavioural disorder go on to develop synucleinopathies^15^. While the connection remains unclear, there is a bidirectional relationship between sleep and dementia. As such, it may be possible to differentiate and detect PLWD using sleep data.

Although sleep anomalies have been reported within the literature, studies that have investigated sleep and dementia have several limitations. Most studies have been conducted in lab environments, which are not natural sleeping environments (i.e., this may disrupt a persons natural sleeping patterns and lead to inaccurate results), or have ascertained sleep quality via sleep assessment questionnaires such as the Pittsburgh Sleep Quality Index^8,9^. Self-reported data may not be accurate^16,17^. Additionally, most studies are conducted over a single night and do not capture long-term variations^10^. Furthermore, using diagnostic tools such as polysomnography (PSG) to monitor sleep is expensive, time-consuming, labour-intensive, potentially distressing for those in the later stages of dementia, and not feasible in the context of large-scale monitoring^18–20^.

Alternatively, Internet of Things (IoT) technologies can provide continuous in-home monitoring, enabling large-scale collection of real-world data, at relatively low cost. Relative to PSG technologies, this is a non-invasive approach that allows longer-term in-home monitoring. IoT technologies work by connecting devices equipped with sensors (i.e., a bed mat sensor for the nocturnal data collection of physiological signals related to sleep activities) to the internet, enabling the collection and sharing of data for monitoring, analysis, and in some contexts, to inform care management^21^. Analysis of data collected with IoT devices such as under-the-mattress bed sensors may help to identify changes in sleep patterns in dementia and in other neurodegenerative conditions where nocturnal perturbations are prevalent. Nocturnal disruptions, including sleep disorders, have been widely reported in Parkinson’s disease, dementia with Lewy bodies, Huntington’s Disease, and Frontotemporal dementia, amongst others^12,22,23^.

The use of predictive analytics using IoT-derived sleep data is limited within neurodegeneration research. There have been promising works in analysing IoT-derived behavioural data in dementia, as well as for IoT-derived sleep data in other disease contexts^24–29^. However, to our knowledge, there is limited research on the use of passively collected sleep data to digitally screen for dementia and other neurodegenerative disease symptoms.

**Fig. 1:**
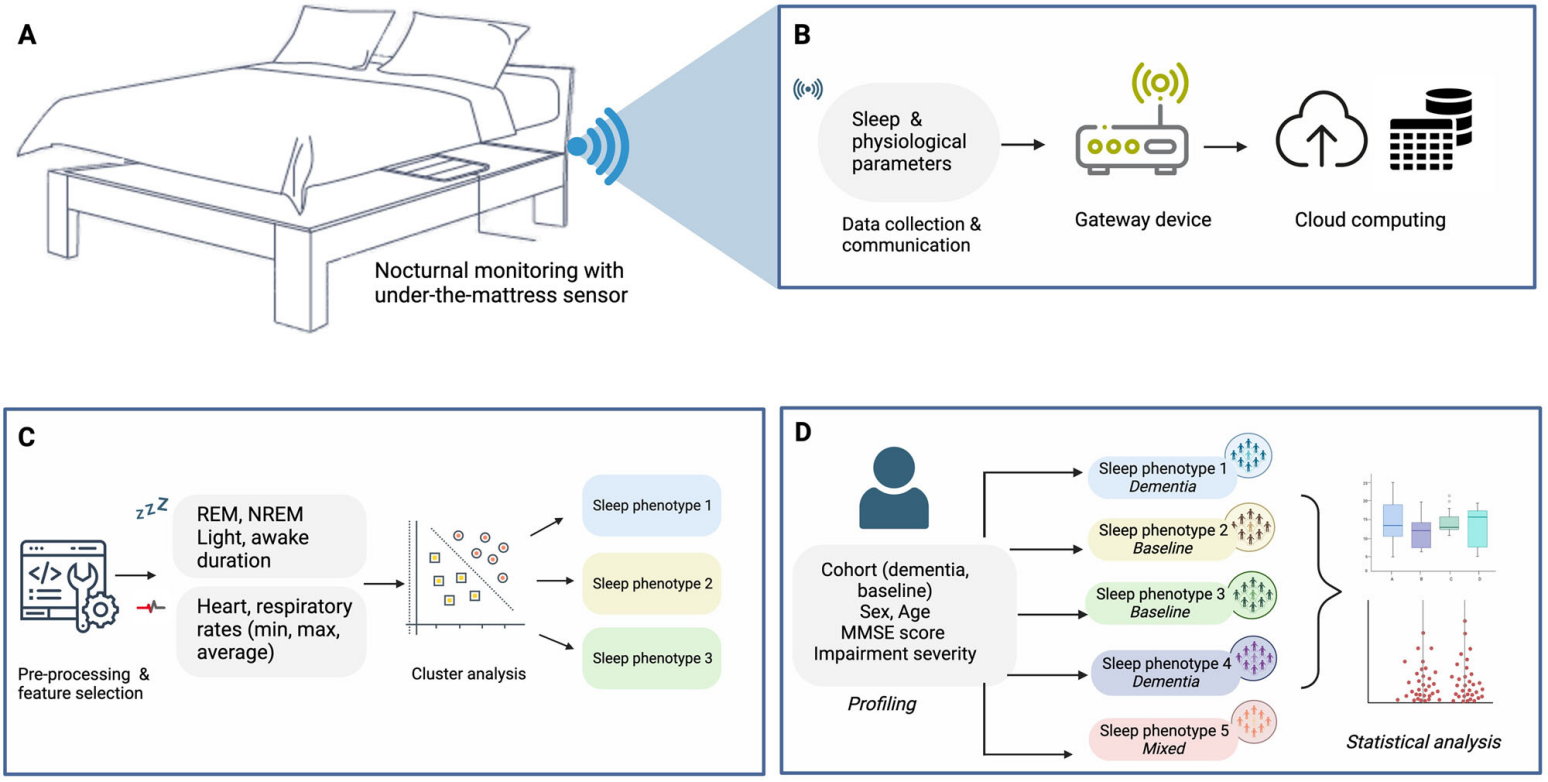
Study overview. The study uses an under-the-mattress sleep monitoring device that passively collects sleep and physiology data (shown in **A**). The data is automatically transmitted via WiFi through a secure channel to a Cloud server where it is anonymised (shown in **B**). The data undergoes pre-processing, design optimisation and feature selection prior to cluster analysis, where sleep phenotypes are identified (shown in **C**). Finally,  profile and statistical analyses are conducted to identify the demographics associated with each grouping and to determine significance between groups (shown in **D**). MMSE Mini-Mental State Examination, NREM non-rapid eye movement, REM rapid eye movement.

In this context, we aimed to demonstrate how IoT-derived sleep data could be leveraged to screen for architectural sleep changes. Employing dementia as a use case, we sought to develop an unsupervised clustering model that could distinguish between individuals with an established diagnosis of dementia or MCI and the general population, based on their longitudinal sleep data. A schematic overview of this study is provided in Fig. 1.

## Methods

### Study design and population

This study was conducted in collaboration with Imperial College London and Surrey and Borders Partnership NHS Trust. It includes two datasets; Minder, an ongoing project since 2018 collecting in-home data from people with an established diagnosis of dementia or MCI at the UK Dementia Research Institute (UK DRI) Care Research & Technology Centre (*n* = 117), and a dataset obtained from participants from the general population using the Withings sleep mat monitoring device (*n* = 5580)^30^. In this paper, the latter is referred to as the General Population dataset.

The General Population cohort comprises individuals from the general population who provided informed consent for their data to be shared. To ensure a representative distribution of the general population and account for the prevalence rates of dementia and MCI^31,32^, we employed a bootstrapping resampling technique. This involved randomly selecting 50 unique participants across 5 folds while considering the distribution of ages within the study group.

The sensor used for this study has been shown in a PSG sleep laboratory study to accurately estimate cardio-respiratory metrics during sleep^33^, where the agreement between the pneumatic sensor and the PSG for respiratory rate (RR), heart rate (HR), and their variability was quantified using Bland-Altman analysis. An overview of the sensor used in this study is provided by Yang and colleagues^33^. The sensor has been validated for the detection of moderate-severe sleep apnoea syndrome, wake after sleep onset (WASO), total sleep duration and sleep efficiency, and quantifying sleep/wake^34–36^. Initial validation studies have been conducted in smaller populations for sleep staging using epoch-by-epoch classification^37^.

Eligible study participants for Minder included adults older than 50 years old clinically diagnosed with dementia or MCI as well as current or previous treatment at a psychiatric unit.

Capacity to consent was assessed in accordance with the Good Clinical Practice, as detailed in the Research Governance Framework for Health and Social Care (Department of Health 2005). Participants lacking the capacity for informed consent were required to have a partner or caregiver who had known them for at least 6 months and was able to attend research assessments with them.

Exclusion criteria were as follows: (1) patients receiving treatment for terminal illness, (2) the presence of severe mental health conditions including depression, anxiety, psychosis, and agitation, and (3) the presence of active suicidal thoughts. In total, 117 participants were selected for participation using the above-mentioned recruitment process. General population participants were all users with a Withings sleep mat^30^ who consented to their data being used as part of the Withings research programme. Demographics of the Minder and General Population participants are provided in Table 1, reporting both the original dataset demographics and those of the processed versions used in the study analysis. Male and female assignments were self-declared by participants. Additional demographic information, including primary diagnoses and Mini-Mental State Examination (MMSE) scores used to assess impairment severity for Minder participants, is provided in Tables 2 and 3. The MMSE scores and their corresponding impairment severity classifications follow the guidelines of the National Institute for Health and Care Excellence^38^.

**Table 1:** Cohort demographics

Cohort	Dataset	Sample size (*n*)	Sex (Male, Female)	Age range
Minder	Original	117	M = 60, F = 57	52–98
Minder	Processed	50	M = 29, F = 21	60–98
General Population	Original	5580	M = 4480, F = 995	19–98
General Population	Processed	50	M = 40, F = 10	60–98

### Data collection

The full study General Population dataset includes over 13 million nights of sleep from June 2020–March 2021 across 5580 participants. The full study data from Minder is also collected from an under-the-mattress sleep sensor, across 117 participants, and is ongoing since 2018, with >14,000 person-nights of data. Table 2.

**Table 2:** Primary diagnosis and MMSE scores in Minder cohort. Scores given to 2.d.p

Primary diagnosis	% of Minder cohort	MMSE score
Alzheimer’s	55.0	13.32 ± 5.92
Vascular dementia	15.0	21.83 ± 3.54
Mixed dementia	7.5	7.00 ± 7.55
MCI	10.0	25.75 ± 5.97
Frontotemporal dementia	2.5	29.00 ± 0.00
Other	10.0	27.50 ± 1.73

*MCI* mild cognitive impairment, *MMSE* Mini-Mental State Examination.

### Statistics and reproducibility

The Minder and General Population datasets were analysed together to determine differences between dementia and the general population using unsupervised learning. Four 30-day period samples across 120 days of sleep data were selected for analysis from each individual in order to provide a representative sample of an individuals sleep patterns over time and reduce short-term variability. 120 days were selected between 31/10/2022 and 01/03/2023 for Minder, and 31/10/2020 and 01/03/2021 for General Population participants. We used the following raw features as input; NREM, REM, light and WASO duration, and the minimum, maximum and average RR and HR, as these features have been reported to be affected in PLWD^8–10^.

**Fig. 2:**
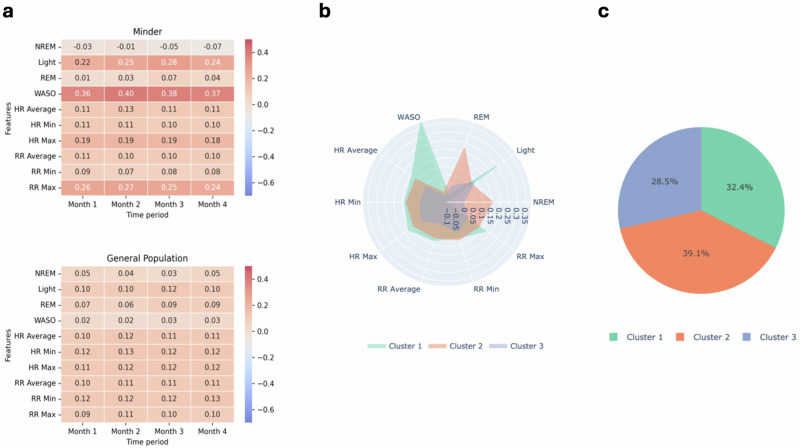
Median features by cohort over 30-day periods, cluster distributions and sample sizes. Visualisations of **a** scaled median feature per cohort (Minder (*n* = 50) General Population (*n* = 50)) per 30-day period, **b** feature distribution per cluster, as determined by K-Means modelling (*k* = 3), and **c** the percentage sample size per cluster. HR heart rate, Max maximum, Min minimum, NREM non-rapid eye moment, REM rapid eye movement, RR respiratory rate, WASO Wake after sleep onset.

The median of each feature was calculated for each user ID across four 30-day period samples across the 120-day period. To minimise the impact  of missing data, participants who had 40% or more missing data (12 or more days) in any 30-day period were excluded from the analysis. From this, 50 participants remained across four 30-day periods in Minder, allowing for 200 data samples. Remaining participants with missing values were imputed with the rolling mean in order to maintain the temporal structure of the data. Figure 2 presents the median features by cohort over 30-day periods, cluster distributions and sample sizes. The General Population sample size was matched via k-fold analysis to allow for equal sample sizes, whereby 50 unique individuals were selected without replacement across 5 folds and combined with the processed Minder dataset for analysis to ensure robust and stable clusters (combined 400 data samples). Table 3.

**Table 3:** MMSE scores and impairment severity

MMSE score Impairment severity	
27–30	Normal cognition
21–26	Mild
10–20	Moderate
0–9	Severe

*MMSE* Mini-Mental State Examination.

### Ethics approval and data anonymisation

The Minder study received ethical approval from the London-Surrey Borders Research Ethics Committee; TIHM 1.5 REC: 19/LO/0102; IRAS: 257561; ISRCTN71000991^39^. All participants in this study either provided their own written informed consent or, if deemed unable to do so, consent was obtained from their legal representatives or individuals with power of attorney. For participants lacking capacity, consent was obtained through their legal representatives after discussions with family members and clinicians to ensure that participation was in their best interest. This process, along with the authority of the legal representatives or power of attorney holders, were reviewed and approved by the ethics committee.

The data collection approval for data collection from the under-the-bed mattress sensor in the Minder clinical study was granted by a clinical IRB panel (IRAS ID: 257561). The anonymised General Population  dataset was provided by Withings (https://www.withings.com) based on a data-sharing agreement for research with Imperial College London in accordance with the applicable security standards and regulations. Information governance and impact assessment approval for storing and processing data from Withings sleep mat data was obtained via from an NHS Information Governance panel (Surrey and Borders Partnership NHS Foundation Trust, reference number: 20201009-DRI). Withings is certified in ISO 27001 and Health Data Hosting (HDS) which comply with the same level of security as health professionals. For the General Population  dataset, consent to obtain user data was previously obtained by Withings. The data extracted for this study was fully anonymised, so re-consent was not required. Withings data collection is in compliance with the GDPR. Part of the Withings data privacy policy includes a legitimate interest, which means pursuing the essential mission of the Data controller (Withings  processes non-identifying data to improve research on the basis of the legitimate interest). This is included in their data privacy statement (https://www.withings.com/uk/en/legal/privacy-policy). User consent is collected in specific cases, and users may withdraw consent at any time.

### Analysis platform

As the data was continuous and processed into more than 3 groups, ANOVA models and Tukey’s post-hoc for multiple comparisons of means were determined to be the appropriate choice of statistical tests. All data analyses, alongside modelling, were conducted using Python 3.9, using the Pandas^40^, Numpy^41^, Scikit-Learn^42^, SciPy^43^ and Pingouin^44^ libraries.

### Model development

Clustering, an unsupervised learning technique, can be used to organise data into different groups based on similarities between subjects^45^. In the context of this work, employing clustering techniques enables unsupervised partitioning of the data based on the processed nocturnal data collected from the under-the-mattress bed sensors. Further analysis of this modelling was conducted to reveal the defining features and subject demographics associated with each grouping, thereby allowing for an understanding of sleep-based differences between PLWD and the general population.

Following k-fold validation, the combined Minder/General Population data was scaled using the z-score and normalised prior to modelling. As clustering algorithms can be broadly classified as centroid-based, distribution-based and hierarchical^45^, for comparison of different algorithms that best classify the input data, one of each was selected. K-means was chosen as the centroid-based model, a Gaussian Mixture Model (GMM) as the distribution-based model, and Agglomerative clustering model as the hierarchical model. The performance of each algorithm was comparatively evaluated using intrinsic evaluation metrics such as the Silhouette Coefficient (SC) score, Calinski Harabasz (CH) and Davis Bouldin (DB) indices, as these do not require ground truth labels.

The optimal number of clusters was determined using the SC scores, DB and CH index scores. Following model optimisation, the model was fitted to the combined input data. A subsequent profiling of the clusters was carried out. As clustering test re-test analysis may assign cluster labelling to the same group across folds, the labels were assigned and mapped across the five folds by calculating the Euclidean distance between the cluster centroids and the origin. Cluster labels were sorted in ascending order and labelled accordingly. The clinical profile of General Population participants is not known, but due to a large dataset, bootstrapping and K-fold sampling, we assume this will limit the effect of comorbidities on sleep architecture. This allows us to conduct a proof-of-concept study to determine sleep-based differences between the two cohorts.

### Profile analysis and statistics

The reported profile analysis reflects the average (±SEM) across five folds. A statistical analysis (ANOVA, Tukeys post-hoc and independent *t*-tests) for the features in each cluster were computed to reveal the defining features and subject demographics underlying each grouping. Cluster descriptions and corresponding demographics (group (mean percentage Minder, General Population ± SEM), sex (mean percentage female, male ± SEM), age (mean ± SEM)) were calculated as percentages of their clusters.

### Reporting summary

Further information on research design is available in the Nature Portfolio Reporting Summary linked to this article.

## Results

### Algorithm selection

To determine a clustering algorithm that optimally clusters the combined Minder/General Population input data, we examined the performance of K-Means, GMM and Agglomerative clustering techniques via several intrinsic evaluation metrics. We selected sleep parameters that are commonly impoverished in PLWD as input features for the model. The features included; NREM (otherwise known as *deep sleep*), REM, light sleep and WASO^46^, as well as the minimum, maximum and average respiratory rate RR and HR. Here, the median of each feature was calculated for each user ID across four 30 consecutive day periods, from the 120-day sample with less than 40% missingness. The algorithms were compared using the SC, and the CH and DB indices as these do not require true cluster labels. All experiments were run 15 times, each with bootstrap samples of the data to calculate confidence interval estimates of the metrics. This allowed us to generate reproducible results and measure the change of metrics between runs.

Based on these metrics, we found that the best-performing model was K-Means with *k* = 3, shown in Table 4. For a comparison of *k* ∈ [2, 25] across models, see Supplementary Fig. 1 and Supplementary Methods. Visualisation of the feature distributions per cluster is shown in Supplementary Fig. 2 and in Supplementary Methods.

**Table 4:** Performance metrics and their mean scores for evaluation of K-Means, GMM and Agglomerative clustering, at *k* = 3 across 15 runs

	SC Score	CH index	DB index
K-Means	0.18	334.06	1.77
GMM	0.12	240.70	1.96
Agglomerative	0.14	227.49	1.95

Higher SC and CH scores indicate better clustering performance, whilst a lower DB score indicates better performance. Results given to 2.d.p.

*CH* Calinski-Harabasz, *DB* Davies Bouldin.

Unsupervised clustering and profiling analysis showed three distinct clusters, including one predominantly composed of people PLWD. This cluster was characterised by increased light and WASO and reduced REM and NREM sleep duration, as reported in Fig. 2. These findings suggest distinct dementia-related sleep phenotypes and support the potential of passive digital technologies for monitoring sleep changes and disease progression in PLWD, and for broader clinical monitoring.

### Clustering description and statistical analyses

**Fig. 3:**
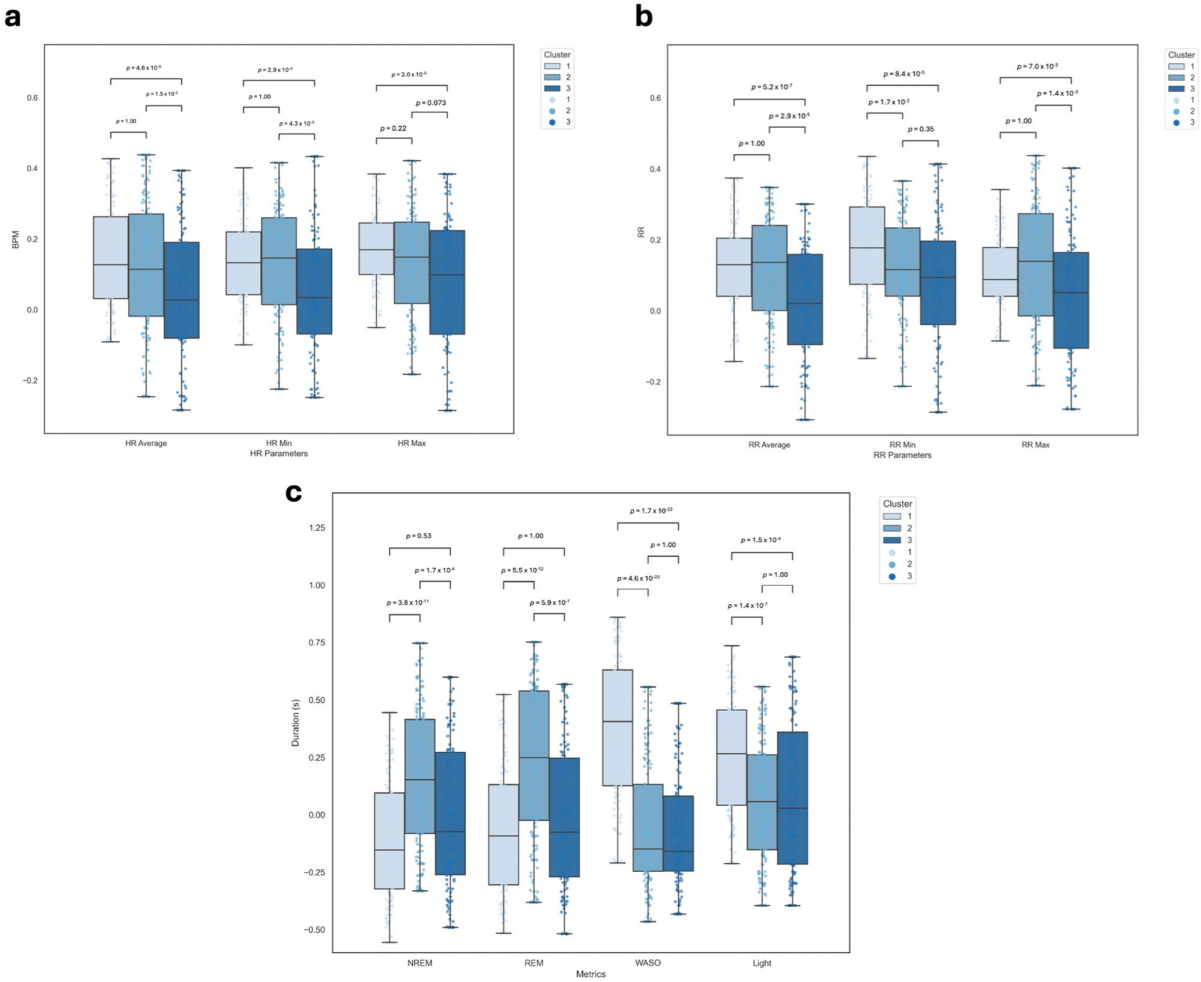
Statistical analysis of heart rate, respiratory rate, and sleep phase metrics across clusters. Analysis of **a** HR parameters (minimum, average, maximum), **b** RR parameters (minimum, average, maximum) and **c** sleep phase metrics (REM, NREM, light sleep, WASO) across clusters (cluster 1; *n* = 49, cluster 2; *n* = 33, cluster 3; *n* = 18), generated by K-Means clustering (*k* = 3). Data was winsorised to account for outliers and scaled via z-normalisation. Error bars denote the SEM standard error of the mean. Statistical significance (independent *t*-tests) is indicated  using horizontal bars. BPM beats per minute, HR heart rate, Max maximum, Min minimum, NREM non-rapid eye moment, REM rapid eye movement, RR respiratory rate, s seconds, WASO wake after sleep onset.

The percentage sample size remains relatively evenly split amongst all clusters; 28.5% has been allocated to cluster 1, 39.1% to cluster 2, and 28.5% to cluster 3. To determine the defining features of these clusters, a statistical analysis between features and clusters via Analysis of Variance (ANOVA) models was conducted (Fig. 3). See Supplementary Data 1 and 2 for the multiple pairwise comparisons between sleep phase metrics and physiological parameters and clusters.

The data is represented in three clusters, each defined by different medians of the sleep parameters across the 120-day selected period. Cluster 1 is defined by a high HR and RR parameters relative to cluster 3, higher WASO and light sleep relative to clusters 2 and 3, and lower REM and NREM mean sleep duration relative to cluster 2. Cluster 2 is similarly defined by higher minimum and average HR parameters and a higher minimum RR relative to cluster 3. It is also characterised by higher NREM and REM mean sleep duration and lower WASO and light sleep. Cluster 3 is characterised by lower HR and RR parameters and low REM, NREM, light sleep and WASO (total sleep duration). The results of a two-way repeated measures ANOVA between clusters and sleep metric are presented in Supplementary Table 1.

### Profile analysis

The majority of Minder participants (PLWD) were grouped into cluster 1 (72% ± 3.22 of the cluster, *p* < 0.001). Cluster 1 was defined by higher HR and RR parameters, higher WASO and light sleep relative to both clusters 2 and 3 and lower REM and NREM mean sleep duration relative to cluster 2 (*p* < 0.001). Cluster 1 showed a higher mean age relative to both clusters 2 and 3 (77.96 ± 1.00, *p* < 0.05). Significantly more females (*p* < 0.05) were clustered in cluster 1 relative to clusters 2 and 3.

**Fig. 4:**
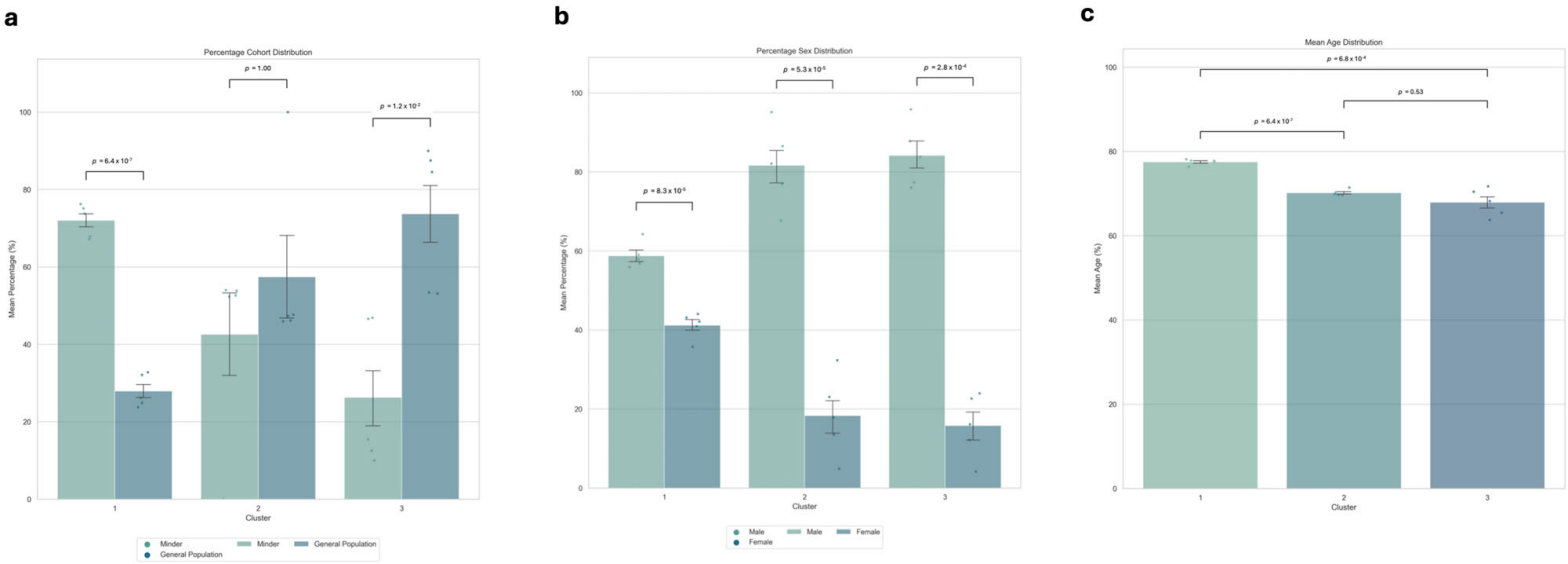
Statistical analysis of average cohort, sex, and age distributions across clusters. Analysis of **a** mean percentage cohort (Minder, General Population) distribution across clusters, **b** mean percentage sex (male, female) distribution across clusters and **c** mean age distributions across clusters where cluster 1; *n* = 49, cluster 2; *n* = 33, cluster 3; *n* = 18. Error bars represent SEM. Statistical significance (independent *t*-tests) is indicated  using horizontal bars. *SEM* Standard error of the mean.

The majority of General Population participants were classified into cluster 3 (66% ± 4.82, *p* < 0.05). Cluster 3 was characterised by lower HR and RR parameters and lower REM, NREM, light sleep and WASO. Cluster 2 showed a lower mean age relative to cluster 1 (71.86 ± 0.74, *p* < 0.05). No age difference was found between cluster 2 and 3. Cluster 2 did not report a difference in the mean percentage split between Minder and General Population participants, showcasing a relatively even split (General Population = 54%, Minder = 46% ± 4.31). Cluster 2 was similarly defined by higher minimum and average HR parameters and a higher minimum RR relative to cluster 3. It was also characterised by higher NREM and REM mean sleep duration and lower WASO and light sleep (*p* < 0.001 and *p* < 0.01) (Table 5, Fig. 4).

**Table 5:** Profile analysis of the combined Minder/General Population dataset using K-Means clustering (*k* = 3)

Cluster N.	Group	Sex	Age
	(% cluster)	(% cluster)	Mean ± SEM
	±SEM	±SEM	
1	General Population = 28%	Male = 59%	77.96 ± 0.93
	Minder = 72%	Female = 41%	
	±3.22	±1.46	
2	General Population = 54%	Male = 81%	73.53 ± 0.74
	Minder = 46%	Female = 19%	
	±4.31	±0.91	
3	General Population = 66%	Male = 84%	71.86 ± 1.00
	Minder = 33%	Female = 16%	
	±4.82	±3.61	

*N* number, *SEM* standard error of the mean.

An analysis of the mean Mini-Mental State Examination (MMSE) scores, which is used to determine impairment severity, reported no significant difference between clusters (Supplementary Fig. 3, Supplementary Methods). The results of the ANOVA between MMSE and clusters within the Minder cohort are provided in Supplementary Table 2. Additional profiling of the Minder participants per cluster (primary diagnoses and MMSE scores) is also reported in Supplementary Table 3.

## Discussion

This work investigated how the analysis of IoT-derived sleep data could be used to identify dementia sleep signatures. Leveraging sleep metric and nocturnal physiology data collected from under-the-mattress bed sensors, we designed an unsupervised machine learning model to differentiate between a cohort of PLWD or an established diagnosis of MCI/dementia and the general population.

We derived the median value of each of the 10 features across 120 days of sleep data and performed a cluster analysis using 3 distinct algorithms (K-means, GMM, and agglomerative). We then determined the optimal algorithm was K-means, and the optimal number of clusters being 3 based on standard evaluation metrics and profile analyses. The following profile analysis reported that cluster 1 was mainly Minder participants (72% ± 3.22, *p* < 0.001), defined by higher HR and RR parameters, higher WASO and light sleep, and lower REM and NREM mean sleep duration. Previous studies have reported distinct patterns of sleep impairment^47,48^. This may represent a sleep phenotype specific to dementia. Our findings are consistent with the literature, where marked decreases in REM and NREM sleep, and increases in light and WASO sleep duration, have been described^11,49^. The changes may be due to cognitive impairments associated with dementia. For example, Rauch and colleagues reported that in AD patients, the mean intensity and number of sleep spindles were positively associated with recall ability and autobiographical memory, respectively^50,51^. Additionally, tau aggregates have also been shown to be correlated with a reduction in NREM sleep^11^. It is important to note that the passive sleep sensor has been validated for physiological parameters, sleep/wake, total sleep duration and WASO^34,36^, but only initial validation studies in smaller populations have been conducted for the REM, NREM and light sleep staging measures^37^. Caution should therefore be exercised when interpreting these results. Further studies are required to validate the accuracy of the sleep staging device in larger cohorts. For this reason, we focused on identifying sleep differences between the Minder and General Population cohorts rather than quantifying specific value differences.

Our results also indicate that Cluster 1, where the main grouping is Minder, is characterised by a high maximum nocturnal RR and HR. Studies have suggested potential links between dementia and changes in respiratory and heart rates during sleep, but the exact relationship is not fully understood. These changes may be due autonomic nervous system dysfunction, which is associated with dementia, and may affect nocturnal RR and HR regulation^52,53^. Other plausible explanations include medications prescribed to PLWD, such as antipsychotic medications that have been shown to impact the autonomic nervous system^54,55^. The relationship is likely multi-factorial and necessitates further research^56,57^. Future research should focus on controlling for comorbidities to further investigate the underlying causes of HR and RR sleep pattern changes. Cluster 1 additionally reported the highest mean age of 77.96 ± 0.93. While age was considered during the interpretation and profiling of the clusters, it was not included as a direct feature in the clustering model. As such, it did not influence the clustering outcome directly. However, differences in age distributions across cohorts may influence profiling results. Due to limited sample sizes, age ranges were controlled for rather than using mean ages.

All clusters were shown to be predominately male. This is due to the imbalance in the original data and cannot be used to draw significance. However, there were significantly more females in Cluster 1 relative to both clusters 2 and 3 (*p* < 0.05). This finding is consistent with previous works. Alexander and colleagues evaluated clinical sub-types in AD using electronic healthcare records and unsupervised clustering, reporting a consistent cluster found in three of the four methods employed, composed of predominantly females^58^. This was also found by Landi and colleagues, who employed deep representation learning of electronic healthcare records^59^. Indeed, studies have reported that females are at higher risk of developing dementia^60^. Females are also reported to have a higher life expectancy relative to males^61^. This may partially explain why the cluster that saw the highest mean age also saw the highest percentage of female participants. Interestingly, cluster 2 saw a relatively even Minder/General Population split (Table 5). It may be that the participants are in this grouping due to undetected MCI. For example, a 2019 systematic review reported the incidence per 1000 individuals in general population samples to be 22.5, 40.9 and 60.1 for ages 75–79, 80–84 and 85+, respectively^62^. Further work would be needed to determine this.

To our knowledge, this is the first study using IoT-derived nocturnal data to identify sleep signatures between PLWD and general population participants. IoT technologies provide an inexpensive, scalable model for continuous passive monitoring, making them particularly advantageous for use in the field of dementia, as well as in wider healthcare. Complementary to clinical findings, the results of our clustering suggest detectable dementia sleep signatures, highlighting the potential for IoT-derived data in dementia screening and diagnosis and providing a proof-of-concept by which we might validate the use of such sensors in PLWD.

Understanding the clusters derived from this model could help clinicians and researchers tailor interventions and treatments. For example, if individuals exhibit specific sleep patterns associated with clusters associated with cognitive impairment, targeted interventions aimed at  improving sleep quality in those clusters can be administered. This may allow for earlier dementia intervention and/or tailored intervention.

Current treatments include non-pharmacological approaches, such as sleep hygiene improvements, cognitive rehabilitation and/or occupational therapy and non-cognitive management for pain, agitation, aggression and distress. Pharmacological approaches may also be considered where the appropriate medication is prescribed in accordance with the specific diagnosis, stage of disease and personal medical history^63^. This cluster analysis has also highlighted key areas that need future investigation, such as understanding the underlying mechanisms behind the association drawn between sleep patterns and cognitive impairment and exploring potential links between dementia severity and sleep disruption.

A limitation in this study was data missingness. To minimise the impact of missing data, participants were excluded from the analysis if they had more than 12 days of missing sleep data within any 30-day period. Reasons for missing data may be due to data integration or sensor issues. The participant may also remove the bed mat (i.e., when changing sheets) or decide to disconnect it. Additionally, the Minder sample size used for analysis was relatively small (*n* = 50), 200 data samples. However, the sample represents over 4800 person-nights of data, which offers sufficient data for analysis.

Additionally, we do not have clinical information relating to the General Population dataset, which may affect an individual’s sleep architecture. We also assume that with K-fold sampling using a large dataset the effect of comorbidities and long-term conditions are reduced. Additionally, no sleep-based exclusions were made for the data used in this study. Possible presence of co-morbid health conditions, such as cardiovascular disease, sleep apnoea or other sleep-based disorders, may also independently affect HR and RR sleep patterns^64^. However, in the Minder cohort, 2 individuals presented with a sleep disorder, and both were shown to be clustered in the dementia-dominant cluster. It is also important to note that the main aim here was to demonstrate a preliminary analysis with passively collected longitudinal data that PLWD present with differentiating sleep signatures, relative to a randomly sampled general population cohort. With this in mind, future analyses with complete clinical information would allow for a more sophisticated understanding of the relationship between sleep and dementia.

Furthermore, the participants in the Minder cohort mainly include individuals with moderate cognitive impairments, which limits the generalisation of the findings to early diagnosis. This work is a preliminary proof-of-concept to highlight the feasibility and effectiveness of using passive in-home technologies for sleep and disease monitoring in dementia. Our future work will aim to apply this analysis to participants exclusively in the earlier stages of dementia, and/or in other neurodegenerative conditions.

Our general population data was recorded during the COVID-19 pandemic. During the height of the COVID-19 pandemic, several international surveys^65–67^ and meta-analyses^69–71^ reported very high rates of insomnia symptoms (including difficulty initiating or maintaining sleep) in the global population (15–50%) with rates even higher among those who had reported having COVID-19 (35–75%)^65,68–70^. However, it was noted by Morin and colleagues that participants who had responded to this survey during the early phase of the pandemic had higher rates than those who completed it later^65^. They suggested that these changing rates were indicative of a reduction in perceived stress as individuals acclimated to the stressors of the pandemic. Regardless, the sleep data from the general population being recorded during the COVID-19 pandemic, the profile analysis of our clustering algorithm shows there is still a distinct difference between the general population cohort and PLWD or MCI, with the clusters that are majority PLWD/MCI having higher WASO and light sleep duration, higher mean HR and RR parameters, and lower REM and NREM sleep by comparison.

Future work will involve analysing a longer duration of sleep data to capture more extended trends and fluctuations. A confirmed non-dementia group will additionally validate these initial findings where differences between the general population and PLWD have been observed. Controlling for age distributions across cohorts should be considered to validate our initial profiling result. Additionally, this analysis may be considered beyond the dementia population, where sleep disturbances are risk factors for other conditions, such as psychiatric conditions (depression, anxiety, bipolar disorder)^71^, or cardiovascular disease^72^. Moreover, applying this analysis to other external cohort data will help further validate the findings reported in this study and identify cases of false positives. Besides addressing study limitations and seeking to improve the current model, future work should consider associations between dementia severity and nocturnal disruption.

IoT devices, whilst offering numerous advantages such as cost-efficiency, scalability and real-time monitoring, may also pose privacy risks if not handled correctly, particularly given the lack of standardised regulations in the presently developing IoT industry. Whilst the necessary information governance practices were employed in this study, these concerns should be considered for future works to ensure researchers prioritise robust ethical considerations. This includes safeguarding individuals’ data privacy through encryption and anonymisation, ensuring transparent and informed consent processes, and addressing data ownership and control.

To summarise, we conducted a clustering analysis of IoT-derived sleep data in an effort to discern differences in sleep signatures between the general population and a cohort of individuals clinically diagnosed with dementia or MCI. Consistent with the wider literature, our results suggested the presence of distinct sleep architectures in PLWD. Not only do these results demonstrate the potential for the use of IoT devices in the field of dementia diagnostics, but they also promote the use of in-home monitoring in wider healthcare.

## Supplementary information

Supplementary Information

Description of Additional Supplementary Files

Supplementary Data 1

Supplementary Data 2

Supplementary Data 3A

Supplementary Data 3B

Supplementary Data 3C

Supplementary Data 4A

Supplementary Data 4B

Supplementary Data 4C

Reporting Summary

## Data Availability

Supplementary statistics for the results of a two-way repeated measures ANOVA between Clusters and sleep phase metrics and physiological sleep variables are in Supplementary Data 1 and 2, respectively. Source data for Figs. 3 and 4 is provided in Supplementary Data 3A–C. The source data for Fig. 4 is in Supplementary Data 4A–C.
