# Supplementary material for: Assessment of sleep patterns in dementia and general population cohorts using passive in-home monitoring technologies
Source: Commun Med (Lond). 2024 Oct 31;4:222. doi: 10.1038/s43856-024-00646-0 (PMC11527978; doi:10.1038/s43856-024-00646-0)
Supplement: Supplementary file 1 — Supplementary Information [file 43856_2024_646_MOESM1_ESM.pdf]

Supplementary Materials

1 Supplementary Methods

1.1 Model and k search

Figure 1 shows the results of the model search experiments for $k \in [2, 25]$ across models (Agglomerative, Gaussian Mixture Model and K-Means). All experiments were run 15 times, each with bootstrap samples of the data to calculate confidence interval estimates of the metrics. This allowed us to generate reproducible results and measure the change of metrics between runs. Based on these metrics, we found that the best-performing model was K-Means with k = 3, shown in Table 2. Firstly, we observe that the k-means algorithm performed the best in general across a wide range of cluster numbers, indicated by larger Silhouette and Calinski-Harabasz scores, and a smaller Davies Bouldin score. Using the elbow method,[1] we picked $k = 3$ as our number of clusters, since it was significantly better than $k = 2$ and performed comparably to $k = 4$, $k = 5$, and $k = 6$ when considering the Silhouette score and the Davies Bouldin score.

1.2 Feature distributions

We selected sleep parameters that are commonly impoverished in PLWD as input features for the model. The features included; non-rapid eye movement (NREM) (otherwise known as *deep sleep*), rapid eye movement (REM), light sleep and wake after sleep onset (WASO) as well as the minimum, maximum and average respiratory rate (RR) and heart rate (HR). Figure 2 provides visualistions or the physiological and sleep metric feature distributions as determined by by K-Means modeling (at k=3).

1.3 Two-way Repeated Measures ANOVA

A two way repeated measures ANOVA (Table Supplementary 1) was conducted to determine the significance between clusters and sleep parameters. As this was significant, a subsequent multiple pairwise comparison was conducted to explore this further. These are provided in the supplementary data files.

1.4 Statistical analysis of MMSE scores

The Mini-Mental State Examination (MMSE) is a widely used tool for the assessment, diagnosis, and monitoring of cognitive decline. It represents 30-point questionnaire that tests various cognitive domains. Lower scores indicate worsening cognition.[2] Table 2 and Figure 3 represent a statistical analysis (ANOVA) and profiling of the Minder participants per cluster. No significance was reported between groupings.

2 Supplementary Figures

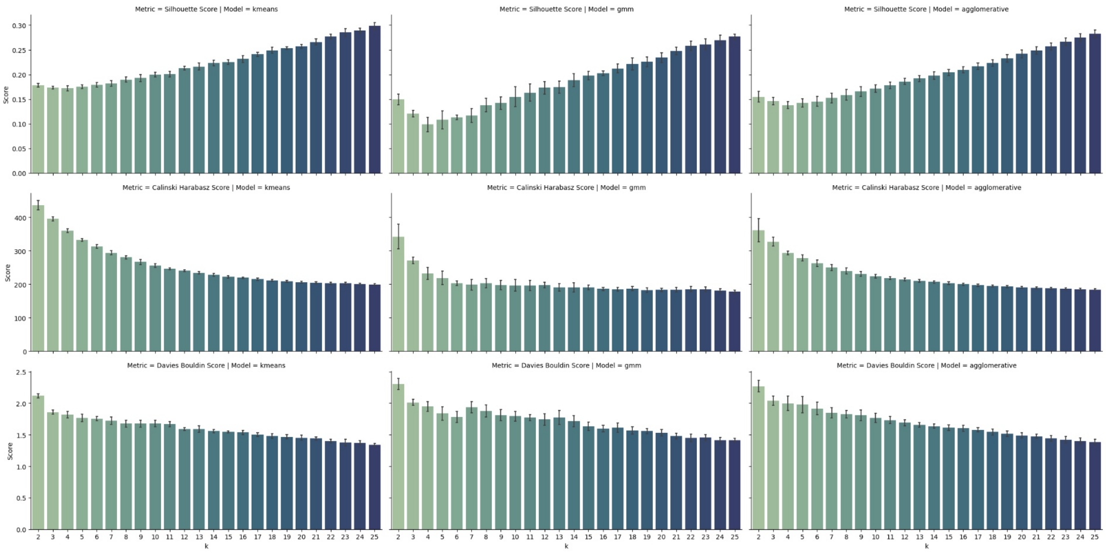

GMM; Gaussian mixture model.

Supplementary Figure 1: Comparison of K-Means, GMM, and Agglomerative clustering algorithms across different performance metrics at varying numbers of $k \in [2, 25]$.

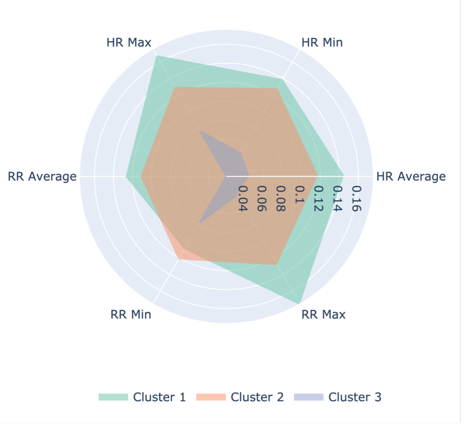

(a) Distribution of sleep parameters

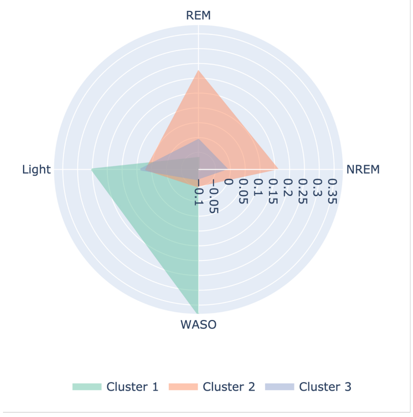

(b) Distribution of sleep phase metrics

HR; Heart rate, Max; Maximum, Min; minimum, NREM; Non rapid eye moment, REM; Rapid eye movement, RR; Respiratory rate, WASO; Wake after sleep onset.

Supplementary Figure 2: Visualizations of (a) sleep parameter feature distribution per cluster, as determined by K-Means modeling (at k=3) and (b) the distribution of sleep phase metrics per cluster.

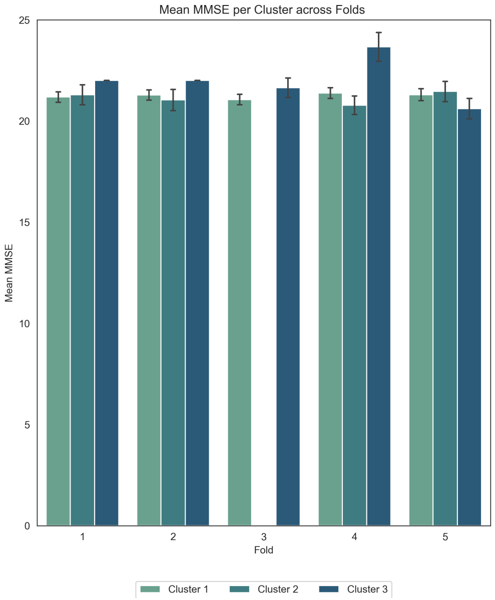

MMSE; Mini mental state examination, SEM; Standard error of the mean.

Supplementary Figure 3: Mean MMSE scores per cluster across folds. Error bars represent SEM.

3 Supplementary Tables

Supplementary Table 1: Results of two-way repeated measures ANOVA.

	Source	SS	DF	MS	F	p-unc	np^2
0	Cluster	$1.39e^{07}$	2	$6.98e^{06}$	0.67	0.51	0.00
1	Metric	$9.16e^{09}$	3	$3.06e^{09}$	295.23	0.00	0.74
2	Cluster * Metric	$7.80e^{08}$	6	$1.30e^{07}$	12.55	0.00	0.20

DF; degrees of freedom, F; f ratio, MS; mean squares, np2; partial eta square effect size, p-unc; uncorrected p-value, SS; sum of squares.

Supplementary Table 2: MMSE ANOVA Results.

Variable	df	sum_sq	mean_sq	F	PR(¿F)
C(ClusterLabel)	2.0	11.87	5.93	0.14	0.86
Residual	3997.0	168674.52	42.20	NaN	NaN

DF; degrees of freedom, F; f ratio, MS; mean squares, np2; partial eta square effect size

Supplementary Table 3: Profile analysis of Minder participants per cluster, including primary diagnoses, mean MMSE, and impairment severity classifications.

**Cluster N.**	**Main grouping**	**n per cluster**	**Minder n per cluster**	**Primary diagnoses (% cohort)**	**Mean MMSE ± SD (severity)**
1	Minder	14	12	Alzheimer's= 27%, Vascular= 50%, Mixed= 67%, MCI= 25%	16.92 ± 6.13 (Moderate)
2	General Population	16	2	Other= 50%	28.77 ± 0.58 (Normal)
3	Minder	17	12	Alzheimer's= 41%, Vascular= 17%, Mixed= 33%, Other= 25%	17.67 ± 8.46 (Moderate)
4	General Population	17	4	Alzheimer's= 9%, Vascular= 17%, Other= 25%	24.00 ± 6.06 (Mild)
5	Mixed	16	9	Alzheimer's= 23%, MCI= 75%, Vascular= 17%	22.22 ± 5.38 (Mild)

MCI; mild cognitive impairment, MMSE; Mini Mental State Examination, N; number, SD; standard deviation.

Supplementary References

[1] Murphy KP. Probabilistic Machine Learning: an Introduction. Adaptive computation and machine learning. Cambridge, Massachusetts London, England: The MIT Press; 2022.

[2] Arevalo-Rodrigue I, Smailagic N, Roqué-Figuls M, Ciapponi A, Sanchez-Perez E, Giannakou A, et al. Mini-Mental State Examination (MMSE) for the early detection of dementia in people with mild cognitive impairment (MCI). Cochrane Database of Systematic Reviews. 2021;27(7):7.
