# Supplementary material for: Assessment of sleep patterns in dementia and general population cohorts using passive in-home monitoring technologies
Source: Commun Med (Lond). 2024 Oct 31;4:222. doi: 10.1038/s43856-024-00646-0 (PMC11527978; doi:10.1038/s43856-024-00646-0)
Supplement: Supplementary file 2 — Description of Additional Supplementary Files [file 43856_2024_646_MOESM2_ESM.pdf]

Description of Additional Supplementary Files

File name: Supplementary Data 1

File description: Multiple pairwise comparison between sleep metrics and clusters.

File name: Supplementary Data 2

File description: Multiple pairwise comparison between physiological parameters and clusters.

File name: Supplementary Data 3

File description: Source data for Figure 3 and 4

File name: Supplementary Data 4

File description: Source data for Figure 4
