# Supplementary material for: Assessment of sleep patterns in dementia and general population cohorts using passive in-home monitoring technologies
Source: Commun Med (Lond). 2024 Oct 31;4:222. doi: 10.1038/s43856-024-00646-0 (PMC11527978; doi:10.1038/s43856-024-00646-0)
Supplement: Supplementary file 11 — Reporting Summary [file 43856_2024_646_MOESM11_ESM.pdf]

nature portfolio

Corresponding author(s): Payam Barnaghi & Louise Rigny

Last updated by author(s): 12.07.24

Reporting Summary

Nature Portfolio wishes to improve the reproducibility of the work that we publish. This form provides structure for consistency and transparency in reporting. For further information on Nature Portfolio policies, see our Editorial Policies and the Editorial Policy Checklist.

Statistics

For all statistical analyses, confirm that the following items are present in the figure legend, table legend, main text, or Methods section.

n/a	Confirmed	
☐	☒	The exact sample size (*n*) for each experimental group/condition, given as a discrete number and unit of measurement
☐	☒	A statement on whether measurements were taken from distinct samples or whether the same sample was measured repeatedly
☐	☒	The statistical test(s) used AND whether they are one- or two-sided<br>*Only common tests should be described solely by name; describe more complex techniques in the Methods section.*
☐	☒	A description of all covariates tested
☐	☒	A description of any assumptions or corrections, such as tests of normality and adjustment for multiple comparisons
☐	☒	A full description of the statistical parameters including central tendency (e.g. means) or other basic estimates (e.g. regression coefficient) AND variation (e.g. standard deviation) or associated estimates of uncertainty (e.g. confidence intervals)
☐	☒	For null hypothesis testing, the test statistic (e.g. *F*, *t*, *r*) with confidence intervals, effect sizes, degrees of freedom and *P* value noted<br>*Give P values as exact values whenever suitable.*
☒	☐	For Bayesian analysis, information on the choice of priors and Markov chain Monte Carlo settings
☒	☐	For hierarchical and complex designs, identification of the appropriate level for tests and full reporting of outcomes
☒	☐	Estimates of effect sizes (e.g. Cohen's *d*, Pearson's *r*), indicating how they were calculated

Our web collection on statistics for biologists contains articles on many of the points above.

Software and code

Policy information about availability of computer code

Data collection	This study includes two separate datasets. The Minder dataset is obtained from Minder, an ongoing study since 2018 (IRAS ID: 257561) collecting in-home data from people with an established diagnosis of dementia at the UK Dementia Research Institute (UK DRI) Care Research & Technology Centre. The data from Minder is collected from an under the mattress sleep sensor. The second dataset, referred to as the General Population dataset, was obtained through a data sharing agreement between the UK DRI Care Research & Technology Centre at Imperial College London and Withings (https://www.withings.com).
Data analysis	All data, statistical analyses and modeling was conducted using Python 3.9, using the Pandas, Numpy, Scikit-Learn, SciPy and Pingouin libraries. The DCARTE library by Dr Eyal Soreq (https://github.com/esoreq/dcarte/) was used for downloading a snapshot of the Minder sleep-mat data.

For manuscripts utilizing custom algorithms or software that are central to the research but not yet described in published literature, software must be made available to editors and reviewers. We strongly encourage code deposition in a community repository (e.g. GitHub). See the Nature Portfolio guidelines for submitting code & software for further information.

Data

Policy information about availability of data

All manuscripts must include a data availability statement. This statement should provide the following information, where applicable:

- Accession codes, unique identifiers, or web links for publicly available datasets
- A description of any restrictions on data availability
- For clinical datasets or third party data, please ensure that the statement adheres to our policy

The data and code that supports the findings of this study are available from the corresponding author upon reasonable request. The DCARTE library by Dr Eyal Soreq (https://github.com/esoreq/dcarte/) was used for downloading a snapshot of the Minder sleep-mat data.

Human research participants

Policy information about studies involving human research participants and Sex and Gender in Research.

Reporting on sex and gender	The subset of the Minder dataset (n=50, >4,800 person-nights) used for analysis included 40 Male participants and 10 Female participants (see above for a description of the datasets). For the General Population dataset, k=5 folds from the dataset was conducted (n=50, >4,800 person-nights in each fold). This was done as sampling with replacement to ensure a representation distribution of the general population. Overall, the dataset had 426 Male participants and 117 Female participants. The average Male/Female split used for analysis was 29 Male participants and 21 Female participants, with a standard deviation of 2.88 and 1.14, respectively. All participants provided written informed consent. A sex based analysis is included as part of the cluster analysis and cluster profiling.
Population characteristics	The research includes two human participant populations. The General Population dataset has a mean age of 70.98 (±5.70) and the Minder population has a mean age of 82.18 (± 7.90). The Minder population, which have confirmed diagnoses of Dementia or mild cognitive impairment (MCI), have the following primary diagnoses: Alzheimer's disease (55% of cohort), vascular dementia (15.0 % of cohort), mixed dementia (7.5% of cohort), MCI (10.0% of cohort), frontotemporal dementia (2.5% of cohort) and 'other' (10.0% of cohort). 'Other' diagnoses: Atypical Early OnsetAlzheimer's (n<=10), Parkinson's dementia (n<=10) and Stroke (n<=10). Those with Stroke as the primary diagnosis were included in the Minder dataset due to having an substantially increased risk of cognitive impairment.
Recruitment	Eligible study participants for Minder included adults older than 50 years old clinically diagnosed with dementia or MCI as well as current or previous treatment at a psychiatric unit. Participants lacking capacity for informed consent were required to have a partner or caregiver who had known them for at least 6 months and was able to attend research assessments with them. Exclusion criteria were as follows: (1) patients receiving treatment for terminal illness (2) presence of severe mental health conditions including depression, anxiety, psychosis, and agitation, (3) presence of active suicidal thoughts. In total, 117 participants were selected for participation using the above-mentioned recruitment process. General Population participants were all users with a Withings sleep mat who consented to their data being used as part of the Withings research program (n=5,580).
Ethics oversight	The Minder study received ethical approval from the London-Surrey Borders Research Ethics Committee; TIHM 1.5 REC: 19/LO/0102; IRAS: 257561; ISRCTN71000991.46 All participants provided written informed consent. The General Population dataset was obtained through a data sharing agreement between the UK DRI Care Research & Technology Centre at Imperial College London and Withings. All data was anonymised prior to use.

Note that full information on the approval of the study protocol must also be provided in the manuscript.

Field-specific reporting

Please select the one below that is the best fit for your research. If you are not sure, read the appropriate sections before making your selection.

☒ Life sciences ☐ Behavioural & social sciences ☐ Ecological, evolutionary & environmental sciences

For a reference copy of the document with all sections, see nature.com/documents/nr-reporting-summary-flat.pdf

Life sciences study design

All studies must disclose on these points even when the disclosure is negative.

Sample size	Due to the following data pre-processing steps to ensure data integrity, the sample size was reduced from n=117 to n=50 for Minder. 120 days of sleep data was selected for analysis in order to provide a representative sample of an individuals sleep patterns over time, and reduce short-term variability. 120 days were selected between 31/10/2022 and 01/03/2023 for Minder, and 31/10/2020 and 01/03/2021 for General Population participants. The median of each feature was calculated for each user ID across the 120-day period. To limit the effect of missing data, participants who had more than 12 days of missing data in any 30 day period were removed from the analysis. Missing values were imputed with the rolling mean in order to maintain the temporal structure of the data. Feature median was then calculated for each user ID across the 120-day period. Random selection via k-fold sampling (k=5) was conducted to take the average findings of the 50 participants

	selected from each fold from the General Population dataset (following the above pre-processing steps), to ensure sample sizes were matched across both cohorts. We determined the sample size to be sufficient based on the number of person-nights this sample size represented (approximately 9,600 person-nights of data).
Data exclusions	To limit the effect of missing data, participants who had more than 12 days of missing data in any 30 day period were removed from the analysis.
Replication	K-fold sampling (k=5) was conducted on the General Population dataset to ensure that findings were reproducible across several 50-person samples. The experiments were repeated five times, where all experimental findings remained consistent across folds.
Randomization	This analysis represents a clustering approach, as such, participants included in the analysis are from two separate and distinct datasets (Minder, General Population). The selected participants within these cohorts are based on the pre-processing steps above. The selection of General Population participants was based on random selection.
Blinding	The investigators were blinded during data analysis. All participants were anonymised, and cluster analysis was performed whereby demographics and experimental group labels were removed prior to modelling.

Reporting for specific materials, systems and methods

We require information from authors about some types of materials, experimental systems and methods used in many studies. Here, indicate whether each material, system or method listed is relevant to your study. If you are not sure if a list item applies to your research, read the appropriate section before selecting a response.

Materials & experimental systems

n/a	Involved in the study
☒	☐ Antibodies
☒	☐ Eukaryotic cell lines
☒	☐ Palaeontology and archaeology
☒	☐ Animals and other organisms
☒	☐ Clinical data
☒	☐ Dual use research of concern

Methods

n/a	Involved in the study
☒	☐ ChIP-seq
☒	☐ Flow cytometry
☒	☐ MRI-based neuroimaging
